# Major Cost Drivers in Assessing the Economic Burden of Alzheimer's Disease: A Structured, Rapid Review

**DOI:** 10.14283/jpad.2021.17

**Published:** 2021-04-24

**Authors:** M. Kosaner Kließ, R. Martins, Mark P. Connolly

**Affiliations:** 1 Health Economics, Global Market Access Solutions Sarl, St-Prex, Switzerland; 2 Unit of Pharmacoepidemiology & Pharmacoeconomics, Department of Pharmacy, University of Groningen, Antonius Deusinglaan 1, 9713 AV, Groningen, The Netherlands

**Keywords:** Indirect costs, healthcare costs, Alzheimer's Disease, societal perspective, economic evaluation

## Abstract

**Background:**

Alzheimer's Disease is the most common cause of dementia, affecting memory, thinking and behavior. Symptoms eventually grow severe enough to interfere with daily tasks. AD is predicted to increase healthcare spending and costs associated with formal and informal caregiving. The aim of this study was to identify and quantify the contribution of the different cost components associated with AD.

**Methods:**

A structured literature review was conducted to identify studies reporting the economic burden of Alzheimer's Disease beyond the healthcare setting. The search was conducted in Medline, Embase and EconLit and limited to studies published in the last 10 years. For each identified cost component, frequency weighted mean costs were calculated across countries to estimate the percentage contribution of each component by care setting and disease severity. Results obtained by each costing approach were also compared.

**Results:**

For community-dwelling adults, the percentage of healthcare, social care and indirect costs to total costs were 13.9%, 17.4% and 68.7%, respectively. The percentage of costs varied by disease severity with 26.0% and 10.4% of costs spent on healthcare for mild and severe disease, respectively. The proportion of total spending on indirect costs changed from 60.7% to 72.5% as disease progressed. For those in residential care, the contribution of each cost component was similar between moderate and severe disease. Social care accounted on average for 85.9% of total costs.

**Conclusion:**

The contribution of healthcare costs to the overall burden was not negligible; but was generally exceeded by social and informal care costs.

## Introduction

**M**any chronic diseases pose significant economic and humanistic burden for patients, families, and society as a whole. For example, it has been estimated that the indirect costs of lost economic productivity of people with chronic diseases are almost 300% greater than the direct costs of healthcare (1). The economic consequences of health-related employment inactivity of people with chronic conditions can also extend to the government due to increased spending on support programs and lost tax revenues (2, 3). Fewer people working, earning income and paying taxes generates lost tax revenue for the government and increasing dependency on public benefits support (4). The externalities of poor health can further extend to family members or friends who may reduce or discontinue their work in order to provide informal care (5, 6, 7, 8). Furthermore, informal caregiving can impact the well-being of those providing care, which is shown to be proportional to the amount of care provided (9, 10) suggesting that as the Alzheimer's disease (AD) population grows, the externalities of the condition also expand.

Researchers have increasingly studied the cost related to informal caregiving due to its significant impact on families as well as the overall contribution to the total economic burden of many chronic conditions (11). Studies have also examined how including the cost of informal care can influence findings of cost-effectiveness studies, where inclusion of the cost of informal care can determine the likelihood that interventions are considered cost-effective or not (12). Many determinants can influence the amount of informal care provided, including age, gender, geographic region, caregiver relationship, the level of dependence of the person requiring care and the amount of social services being provided (1, 13).

The importance of informal caregiving is exemplified by AD, which is a progressive chronic condition with increasing global prevalence (14). AD is a continuum with the first clinically recognizable stage being Mild Cognitive Impairment (MCI) (15). MCI refers to individuals who function similarly to their peers and suffer some cognitive impairment, but it is not sufficiently severe for it to be considered dementia (16). As the disease progresses, symptoms gradually worsen and in the later stages patients typically lose their independence and become dependent on formal or informal care. As a result, AD is predicted to increase healthcare spending and costs associated with formal and informal caregiving compared to an average aging population.

This is particularly important as AD progresses, and more intensive care is required (17, 18, 19). Increasing demands are placed on informal care at a time when the proportion of working aged adults is decreasing in many advanced economies, which could influence economies and labor markets (20). There is growing evidence of the significant economic burden that AD poses on the healthcare system as well as on patients and their families. To further understand the contribution of healthcare costs to overall costs attributed to AD, we have reviewed the literature to identify studies that provide comprehensive estimates of financial burden including productivity losses, informal care costs, institutionalization costs and other economic domains. We believe that dissecting the cost components can give a more complete picture of the overall burden of AD, emphasize the major cost drivers associated with AD, and in the end serve as a foundation for future policy frameworks.

## Study aims

The aim of this literature review was to provide an overview of the different cost components associated with AD and estimate the proportion of overall costs of AD that are attributable to healthcare in comparison with all other attributable costs incurred by individuals, households and society.

## Methods

### Search strategy

A comprehensive search strategy was constructed using controlled vocabulary and free-text terms relating to the population, outcomes and study designs of interest. Population terms included those related to AD and mixed dementia, as well as neurocognitive disorders other than AD, and those defined by the Diagnostic and Statistical Manual of Mental Disorders (DSM-V) and recognized patient societies, in order to reduce irrelevant studies. Outcome terms were clustered around five concepts: labor force participation and income, disposable income, social security, disability allowances and indirect costs. These measures are typically not included in randomized trials; or are reported as secondary outcomes for which studies are not powered to analyze. Additionally, when these data are collected alongside randomized trials they are intervention-specific, restricted to shorter follow-up periods and of limited generalizability due to strict trial inclusion criteria (21). Therefore, a search filter for observational studies formed the last search concept. The search was limited to humans and to studies published in the last 10 years. No language limitations were predefined. The full strategy provided in Supplement 1 was used for searching MEDLINE (PubMed) and adapted for searching EMBASE (OVID) and EconLIT. Backwards snowballing was conducted on eligible studies to identify further relevant research.

### Study eligibility

#### Population

Individuals identified with MCI likely due to AD or AD with or without another form of dementia were included along with their caregivers. Populations limited to a single gender or AD in combination with nondementia health conditions were excluded.

#### Comparison

Comparisons of AD to a cognitively normal population or between different stages of AD were of interest.

#### Outcomes

For the patient and caregiver, the outcomes of interest included direct and indirect healthcare costs; these including but not limited to income, labor force participation, economic (in)activity, work adaptation; disposable income; social insurance allowance or benefit; disability allowance and caregiver's allowance. Studies assessing total societal costs which included health costs and the cost of each component as well as the total were included. However, studies reporting only on a single component of economic impact, e.g., only informal costs or health costs only, were excluded.

#### Study design

Non-interventional, observational studies providing an overview of AD were included. Interventional studies were kept in if they reported relevant outcomes; however, they were of less priority. Randomized or quasi-randomized clinical trials, traditional and systematic literature reviews, qualitative studies, methodological papers or study protocols, economic modeling studies, comments, editorials and letters were excluded. Studies with less than 10 subjects per arm were also omitted.

### Study selection

References were downloaded into ENDNOTE version 9.3. Study titles and abstracts were screened against the eligibility criteria described above by a single reviewer. The full texts of relevant studies were subsequently obtained and screened by two independent reviewers. Posters of conference abstracts were sought if the material had not been published in a journal manuscript. Uncertainties between reviewers were resolved by discussion with a third reviewer.

### Data extraction and synthesis

Data were extracted from each study by a single reviewer on study design and duration, country, care setting, sample size and age, disease diagnosis and disease severity; measurement and costing of resources (costing approach, costing year and currency), and the absolute mean and variance of each cost component and of the total costs. The resource items comprising each cost component were also recorded.

The percentage of total costs covered by each component was calculated for the overall AD population in each study and by disease severity. Outcomes from cross-sectional studies and at baseline from longitudinal studies were narratively synthesized. For each cost component, frequency weighted mean costs were calculated to summarize results across countries by disease severity, and per country when multiple studies were available. For this purpose, all costs were inflated to 2019 using country specific consumer price index values (22) and then converted to Euros. Primary analysis was based on studies that used the human capital approach for valuing indirect costs (23) and repeated for each care setting. When studies reported multiple analyses, results obtained with supervision time from a caregiver or family member were included. A separate assessment was conducted on studies that valued informal care using the labor replacement approach, i.e., by using the cost for hiring a professional caregiver. Results obtained with the two costing approaches for the community setting were compared. Economic elements not included in the estimation of the total societal costs, i.e., income, were narratively summarized.

## Results

**Figure 1:**
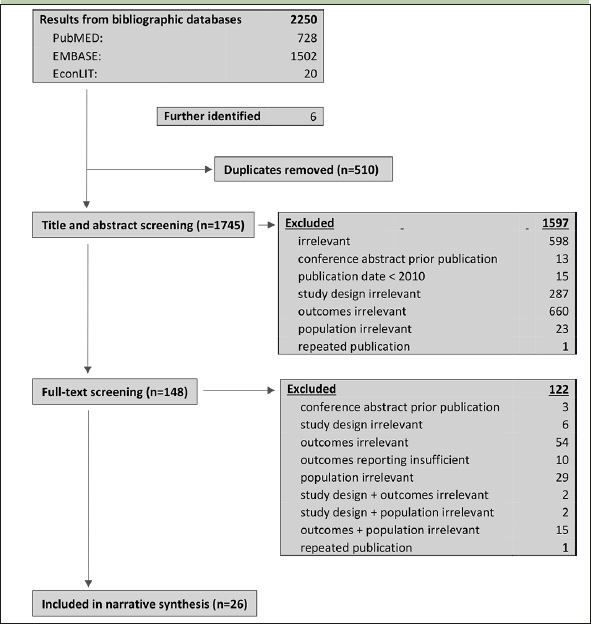
Flow of study selection

The search yielded 2250 results. After removing duplicates, the titles and abstracts of 1740 records were screened of which 143 were considered relevant for full-text screening. Of these, 3 were conference abstracts for which journal publications were identified; 1 was a repeated publication; 10 provided an insufficient description of methods or results and 108 met at least one exclusion criteria. 21 publications were included in a narrative synthesis. Five publications were further included in synthesis after backward snowballing. Study selection is depicted in Figure 1.

### Characteristics of individual studies

Ten publications reported results from the GERAS I (18, 24, 25, 26, 27, 28), GERAS II (29, 30), and extensions of the GERAS to Japan (31) and the USA (32). The remaining 20 publications included the ECO, EVOCOST and Codep-AD studies from Spain (33, 34, 35, 36); the ECAD from Ireland (17, 37); one multinational study (38); a cluster-randomized observational study from China (39, 40); and others from France (41), Germany (42), Sweden (43), and the USA (44, 45, 46, 47, 48). Together there were 17 studies with unique methodologies.

One retrospective case-control study from the USA used a claims database to assess patient and caregiver medical costs in comparison to a cognitively healthy spouse-patient dyad (47, 48, 49, 50, 51). Based on population survey data also from the USA, Ton (46) assessed the relationship between cognitive decline (MCI and AD) and household income in addition to patient medical costs.

The total socioeconomic burden was estimated in 15 studies. The characteristics of these are summarized in Table 1. Two studies used random sampling to identify study sites (33, 39, 40). In the remaining studies, participants were conveniently sampled from their healthcare settings by their local healthcare providers. Longitudinal studies (9 studies) limited their sample to community-dwelling adults, with exception of the ECO study that also included individuals from a residential setting. Three studies further restricted their sample by disease severity: the EVOCOST study focused on adults with moderate disease severity (34); the GERAS-US study (32) compared mild AD against MCI; and Zhu (45) compared adults with MCI against cognitively healthy adults. Cross-sectional studies (6 studies) included a broad sample from the community and residential setting, except for Gervès et al (2014) who studied community-dwelling adults; and most did not specify an age-limit for inclusion (35, 36, 38, 42, 43). Disease severity was defined by the Mini-mental State Exam (MMSE) scores in 14 studies; and by the Clinical Dementia Rating (CDR) in the ECO and Codep-AD studies (33, 35, 36). Discrepancy was observed between studies in the diagnostic criteria for AD and disease staging based on MMSE scores. Two studies staged disease severity by dependency level (36, 44).

**Table 1:** Characteristics of studies assessing total socioeconomic burden of AD

Study	Country	Study design	Follow-up (months)	Care setting	Sampling approach	Sample	Disease definition	Disease severity
GERAS I (18, 24–28)	DE, FR, UK	Longitudinal prospective	18	community	convenience sampling	Age criteria: 55+ yrs n=1497 dyads	probable or possible AD^1^	mild: MMSE 21–26 moderate: MMSE 15–20 severe: MMSE ≤ 15
GERAS II (29, 30)	IT, ESP	Longitudinal prospective	6	community	convenience sampling	Age criteria: 55+ yrs n=578 dyads	probable AD^2^	mild: MMSE 21–26 moderate: MMSE 15–20 severe: MMSE ≤ 15
GERAS-J (31)	JPN	Longitudinal prospective	18	community	convenience sampling	Age criteria: 55+ yrs n=553 dyads	probable AD^2^	mild: MMSE 21–26 moderate: MMSE 15–20 severe: MMSE ≤ 15
GERAS-US (32)	USA	Longitudinal prospective	36	community	convenience sampling	Age criteria: 55+ yrs n=1239 carers, 1237 patients	MQ mild AD^2^	MMSE ≥ 24 & FAQ < 6 MMSE ≥ 20 & FAQ ≥ 6
Codep-AD (35, 36)	ESP	Cross-sectional	NA	community and residential	convenience sampling	Age criteria: None n=343 dyads	probable or possible AD^1^	questionable: CDR 0.5 mild: CDR 1 moderate: CDR 2 severe: CDR 3
Jia (39), Yan (40)	CN	Cross-sectional	NA	community and residential	cluster sampling	Age criteria: 60+ yrs n=2507 carers, 3046 patients	probable or possible AD^1^	For illiterate dementia mild: MMSE 21–24; moderate: MMSE 11–20; severe MMSE ≤10. For literate dementia mild: MMSE 16–19; moderate: MMSE 8–15; severe: MMSE ≤7.
ECAD study (17, 37)	IE	Longitudinal prospective	24	community	convenience sampling	Age criteria: 50+ yrs n=72 patients	MCI3, probable or possible AD^1^	MMSE, not specified
Reese (42)	DE	Cross-sectional	NA	community and residential	convenience sampling	Age criteria: NR n=395 dyads	MQ, probable or possible AD^1^	MCI: MMSE ≥ 26 mild: MMSE 21–25 moderate: MMSE 15–20 severe: MMSE ≤ 15
Mesterton (43)	SWE	Cross-sectional	NA	community and residential	convenience sampling	Age criteria: None n=233 dyads	AD with or without vascular components	mild: MMSE 20–26 moderate: MMSE 10–19 severe: MMSE < 10
ECO study Coduras (33)	ESP	Longitudinal prospective	12	community and residential	stratified multistage probabilistic sampling	Age criteria: 50+ yrs n=560 dyads	probable or possible AD^1^	mild: CDR 0.5 mild to moderate: CDR 1–2 moderate to severe: CDR 3
Michaud (44)	USA	Longitudinal prospective	36	community	convenience sampling	Age criteria: 50–85 yrs n=132 dyads	probable or possible AD^1^	Dependence Scale stages
Gervès (41)	FR	Cross-sectional	NA	community	convenience sampling	Age criteria: 60+ yrs n=57 patients	AD	mild: MMSE ≥ 20 moderate to severe: MMSE ≤ 19
Gustavsson (38)	SWE, ESP, UK, USA	Cross-sectional, multinational	NA	community and residential	convenience sampling	Age criteria: None n=1222 dyads	probable or possible AD^1^	mild: MMSE > 20 moderate: MMSE 10–20 severe: MMSE < 10
EVOCOST study (34)	ESP	Longitudinal prospective	12	community	convenience sampling	Age criteria: NR n=162 dyads	AD	moderate: MMSE 10–19
Zhu (45)	USA	Longitudinal prospective, case-control	36	community	convenience sampling	Age criteria: 55–90 yrs n=259 patients, 107 controls	MCI, cognitively healthy controls	MCI: MMSE ≤ 24, CDR=0.5 Controls: MMSE > 26, CDR=0, GDS 1–2


1. National Institute of Neurological and Communicative Disorders, and Stroke and Alzheimer's Disease and Related Disorders Association criteria; 2. National Institute on Aging and Alzheimer's Association Alzheimer's criteria; 3. International Working Group on Mild Cognitive Impairment (J Intern Med 2014; 256(3):240–246); AD: Alzheimer's Disease. CN: China. DE: Germany. ESP: Spain. FR: France. IE: Ireland. LT: Italy. JPN: Japan. MCI: Mild cognitive impairment. NR: Not reported. NA: Not available. SWE: Sweden. UK: United Kingdom. USA: United States of America. MMSE: Mini-Mental State examination. FAQ: Functional Activities Questionnaire. CDR: Clinical Dementia Rating. GDS: Global Deterioration Scale.

Overall, adults with MCI likely to be due to AD were included in 3 studies (17, 32, 37, 42); their outcomes were reported separately from adults with AD in the GERASUS (32).

All 16 studies included patient health care, social care and informal care in their estimation of total socioeconomic burden. There were minimal differences across studies in the resource items assessed as most studies used the Resource Utilization in Dementia (RUD) (52) or RUD-Lite (53)instruments for measuring resource utilization. The case-control study by Zhu (45) differed from the others by using the Resource Use Inventory (54) to capture resource utilization and not valuing the use of informal care in MCI. It is also noteworthy that Reese (42) conducted their economic evaluation from the perspective of the German statutory health insurance; formal and informal care were assessed together as a component of social care. This evaluation also estimated productivity losses of the patient and caregiver. Productivity loss of the caregiver was evaluated independently from informal care in one other study where informal care was accounted as lost leisure time (35). Informal care was accounted as productivity loss in one study each from the USA (44) and China (39, 40). The Chinese study further considered intangible costs which accounted for 4.2% of total costs. Additionally, healthcare costs of the caregiver were evaluated by GERAS I, GERAS II-Spain and GERAS-US.

The contribution of patient health and social care and indirect costs to total societal costs, without caregiver health care and intangible costs, were calculated across all studies. Indirect costs related to informal care and productivity loss when evaluated separately.

### Cost components by setting

The cost components attributed to the MCI population were obtained from a single study where the largest component of overall costs was patient health care costs (50.9%) followed by informal care costs (40.1%) when using the human capital approach. The case-control study by Zhu (45) found hospitalization to be the largest component of medical costs and that adults with MCI required significantly more informal care than cognitively healthy adults.

In community-dwelling adults with AD, the weighted mean contribution of health care costs was 26.0%, 15.7% and 10.4% for mild, moderate and severe forms of AD, respectively; and averaged 13.9% across all severity levels. Results summarized in Table 2 show that the weighted mean contribution of indirect cost to the overall cost burden was substantially high and increased as disease progressed representing 60.7%, 67.1% and 72.5% for mild, moderate and severe AD, respectively. Country-level data presented in Supplement 2 show that patient health care costs formed a greater component of total costs in the USA compared to European countries at all disease severity levels; and the least in Italy where informal care costs exceeded 80% of total costs. Further, social care costs composed a larger amount of the total costs in Japan and Sweden, and even exceeded the contribution of informal care in Sweden.

**Table 2:** Weighted mean (min-max) contribution of each cost component to total costs across countries

	Community				Residential				Combined Community & Residential			
	n dyads (studies)	Healthcare	Social care	Indirect costs	n dyads (studies)	Healthcare	Social care	Indirect costs	n dyads (studies)	Healthcare	Social care	Indirect costs
MCIt	677 (1)	50.9%	9%	40.1%	-	-	-	-	-	-	-	-
Mild AD	1838 (5)	26.0% (7.8%–36.2%)	13.3% (5.8%–45.3%)	60.7% (30%–82.2%)	-	-	-	-	91 (1)*	14.5%	72.2%	13.3%
Moderate AD	1388 (5)	15.7% (6.3%–30.4%)	17.3% (0%–37.2%)	67.1% (43.2%–84.1%)	132 (1)*	8.2% (5.5%–10.5%)	85.4% (81.7%–92.2%)	6.4% (2.4%–9.9%)	558 (2)*	13.8% (5%–20.8%)	61.3% (47.1%–84.7%)	24.9% (10.3%–40.2%)
Severe AD	1083 (4)	10.4% (2.6%–24.4%)	17.1% (9.3%–47.3%)	72.5% (39.1%–87.8%)	179 (1)*	8.5% (7.3%–9.7%)	86.2% (82.9%–89.6%)	5.3% (2.3%–7.4%)	432 (2)*	10.9% (4.7%–14%)	69.1% (58.7%–59.4%)	20.0% (5.9%–32.9%)
All AD x	3885 (7)	13.9% (4.2%–29.9%)	17.4% (7.4%–42.1%)	68.7% (38%–86.1%)	434 (2)	8.6% (6.8%–9.9%)	85.9% (82.3%–90.8%)	5.5% (2.3%–8.1%)	5104 (5)	23.8% (6.7%–33.7%)	32.4% (15.6%–83.9%)	41.5% (9.4%–67.8%)


† only one MCI study identified.

For adults living in residential care, the weighted mean contribution of cost components was similar between moderate and severe AD, as shown in Table 2. Across severity levels, patient social care formed 85.9% of total costs and patient health care was slightly larger than that of informal care (8.6% vs. 5.5%). Further, the percentage contribution of each cost component was similar between countries. The difference in minimum and maximum values between Germany, Spain, Sweden, UK and USA were 3.1%, 8.5% and 5.8% for patient health care, social care and indirect costs, respectively between Germany, Spain, Sweden, UK and USA. Country-level data are tabulated in Supplement 2.

In studies that assessed both community and residential care settings, the percentage contribution of cost components varied between countries in terms of social care (15.6%–83.9%) and informal care (9.4%–67.8%). Looking at country-level data (Supplement 2), this outcome was heavily influenced by high social care costs and little informal care in Sweden. Additionally, social care constituted a smaller component of total costs than patient healthcare in China (15.6% vs. 32.5%) than in European countries.

### Comparison of costing approaches

**Figure 2:**
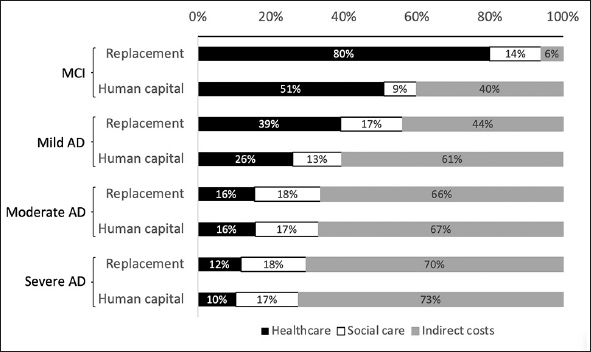
Comparison of labor replacement method and human capital approach for valuing indirect costs and influence on percentage contribution of each cost component

The choice of method for costing informal caregiving time had a substantial impact on the distribution of cost components in the early stages of cognitive decline. Using the labor replacement approach increased the weighted mean contribution of patient healthcare to total costs for MCI (79.9% vs. 50.9%) and mild AD (39% vs. 26%), as shown in Figure 2. Country-level results provided in Supplement 2 show that this was especially true for the USA where the contribution of patient healthcare almost doubled (36.2% to 65.4%). Smaller, but observable changes also occurred in Spain, Germany and Italy. Data for these countries came from analyses that excluded supervision time from informal care. Additional analysis was carried out using results from the GERAS studies to explore how the inclusion of supervision time influences results. Across France, Germany, UK, Spain and Italy, the weighted mean contribution of patient health and social care were equally elevated by 5% to 6% with the exclusion of supervision time from informal care calculations. Results are presented in Supplement 3.

### Contribution of caregiver healthcare to overall costs

Across the GERAS-I countries, Spain and the USA, caregiver healthcare costs accounted for 6.9% of total costs in adults with MCI likely due to AD (32) and 3.7% of total costs in those with AD. As shown in Table 3, the percentage contribution of this component to the total cost decreased substantially from mild (11.5%) to moderate AD (4.4%) and reached 2.3% for severe AD. Across AD severity levels, the contribution of caregiver healthcare costs showed little variation between countries (3% – 4.2%).

**Table 3:** Weighted mean (min-max) contribution of caregiver healthcare costs

	n	Patient HC	Patient SC	Caregiver HC	Informal care
MCI	677	39%	23.4%	6.9%	30.7%
Mild AD	1333	23.7% 8.2%–24.2%	10.3% 6.2%–18.2%	11.5% 3%–16.12%	54.5% 62.4%–76.3%
Moderate AD	590	13.1% 7.7%–18.7%	15.1% 8.3%–19.8%	4.4% 2.4%–6.9%	67.5% 62.9%–72.1%
Severe AD	604	10.2% 5.5%–13%	14.7% 9.1%–21.9%	2.3% 1.8%–3.8%	72.8% 67.9%–76.1%
All AD	1877	12.2% 7.2%–17%	14.3% 8.2%–18.4%	3.7% 3%–4.2%	69.8% 66.7%–71.3%


AD: Alzheimer's Disease. MCI Mild Cognitive Impairment. HC: Healthcare. SC: Social care.

### Impact of AD on other socioeconomic aspects

Ton et a (2017) (46) demonstrated that in the USA not only adults with AD but also those with MCI had greater medical expenditure and less household income than cognitively healthy adults (<0.001). This result remained highly significant after adjusting for age, sex, race, education, marital status, residential region and comorbidities (<0.015). Another study demonstrated that, compared to MCI, significantly more individuals with mild AD were pushed to an income below the federal poverty level. Patients' employment rates were found to significantly drop from 21.4% to 9.4%; and the number of employed adults who reduced their work significantly rose from 3.2% to 13.8% (32). In the broader AD population, a significant relationship between dependency and household income has not been found (44).

When examining the impact of AD on household expenditure (47, 48), an US study indicated that annual health care costs were double the amount of costs of a cognitively healthy household ($6,028 vs. $2,951). Patient health care costs were significantly higher than age, sex and comorbidity-matched adults ($4408 vs. $1473, p<0.001). Spousal caregivers accumulated significantly higher costs for AD-related and mental health prescription; but on average were not significantly different from spouses of cognitively health adults.

## Discussion

The rising costs of treating AD and the impact on households and caregivers has been a topic of concern for researchers, policy-makers and planners for many years (55). The work described here helps to put expenditure into perspective to understand major cost drivers in the delivery of care to people with AD. This review has illustrated that in community-dwelling adults with AD, patient healthcare costs constitute the smallest component of the total cost burden representing, on average, 13.9% across all AD severity levels. Furthermore, the contribution of healthcare costs to the overall cost burden decreases as disease progresses and as informal care needs increase. As described here, the costs of informal care represent approximately 60% of total costs, and reach 72.5% of the total cost burden in severe AD. The difference between the contribution of patient healthcare and indirect costs was substantially reduced in early stages of AD when using the replacement labor approach to valuing informal care. This may be due to higher employment rates of the caregiver of adults with MCI and mild AD compared to the later stages; and that this is disregarded with the use of a uniform cost to value caregiving time. Robinson (32) reported employment rates of 48.3% and 43.4% respectively for patients with MCI and mild AD; with later stages of AD this tends to drop below 30% (18, 30).

Variation in the distribution of the cost components in the community and residential professional care settings emphasize the importance of studying each setting separately. When costs were pooled across settings, results were heavily influenced by residency care and showed high variability between countries. It is important to put the informal care costs into perspective as these represent lost earnings for individuals with significant economic consequences (56). Therefore, interventions that delay progression can offer economic benefits due to reduced need for informal and formal care.

We observed that the distribution of cost components was relatively similar between European countries. In Italy, however, there was a heavy reliance on informal care and little utilization of medical care which became even more apparent with increasing disease severity. The provision of long-term care by the family may be due to differences in the formalization of and access to healthcare compared to other European countries (29). The greater contribution of community care in Japan, compared to European countries, may be due to the caregiver being an adult-child of the person with AD (31), and in Sweden due to the availability of different social care structures (38). Such factors have been considered in other comparisons of country-level data (24, 26).

This review identified few studies evaluating the broader economic burden of MCI likely due to AD, probably because of the recent introduction of this term and the difficulty to establish this diagnosis (57). These studies demonstrated that individuals with MCI likely due to AD require social care and informal care more than their age-matched peers; and that this is further increased in those with mild AD dementia (32, 45). A similar trend is seen with caregiver health care costs when they are included in the estimation of total costs. These results highlight the importance of reporting disaggregated outcomes across early stages of cognitive decline. As more sensitive diagnostic methods become available to detect changes in cognition and more therapies become available to slow down progression early in the AD continuum, the need to explore the wide socioeconomic impact of cognitive decline will become more pertinent.

The results of this review should be interpreted with caution as a small number of studies were included. A larger number of studies might have been identified by removing the search limit on publication dates. The intention of this search limit was to identify studies reflecting current treatment practices. As part of a rapid review, study screening and data extraction were carried out mostly by a single reviewer, and the quality of the included studies were not assessed due to limited time and resources. The exclusion of quality appraisal is justifiable as a meta-analysis of study results was not possible. The analysis was nonetheless quantitative in nature and would not have benefited from the inclusion of qualitative evidence. Calculation of a frequency-weighted mean cost across countries was seen as a descriptive method for summarizing estimated costs per person. Differences in criteria for disease diagnosis and staging were not considered in data synthesis. Only the extensions of the GERAS study applied the more recent diagnostic criteria from the National Institute on Aging and Alzheimer's Association Alzheimer's (NIA-AAA) (58). Study-level results differed more between diagnostic criteria than between disease staging based on MMSE scores. Differences in AD severity categorization are likely to generate cost data somewhat different in absolute terms. There is a clear trend in the data showing that a reduction in the proportional contribution of healthcare costs is accompanied by an increase in the contribution of indirect costs, as severity progresses (Figure 2). The authors believe that this overall trend is unlikely to be substantially altered were AD categories more homogeneous.

NIA-AAA criteria distinguish AD dementia from earlier stages of cognitive decline, not limited to memory loss alone, and from other dementing conditions. They also recognize the additional use of imaging methods or biomarker analysis in increasing certainty in diagnosis, particularly for the differential diagnosis of MCI likely due to AD. However, at time of publication ancillary testing was described as optional clinical tools, advocating more investigational research on their use and standardization (57, 58). The Alzheimer's Disease Neuroimaging Initiative has played an important role in the quest to find sensitive biomarkers and diagnostic tests; and have developed standardized methods for clinical tests, magnetic resonance imaging, positron emission tomography and cerebrospinal fluid biomarkers (59). Multi-modal use of neuroimaging and biological markers has been recommended as the way forward for detecting changes in cognition throughout the AD pathophysiology (60), and for predicting future decline (59). Blood biomarkers have also been developed as a non-invasive, low-cost alternative to cerebrospinal fluid biomarkers; and have shown to be effective in differentiating AD, MCI and cognitively normal controls (59, 61). These recent advances will likely impact the incidence of MCI due to AD and AD dementia and their associated health care costs. Study-level results from this review suggest the contribution of patient health care costs to be lower and that of social care costs to be higher with NIA-AAA criteria compared to older diagnostic criteria. Future observational studies reflecting the use of modern methods are needed to explore this hypothesis.

## Conclusions

Healthcare costs can cover up to 30% of the overall burden of AD; but is generally exceeded by the costs associated with social care and informal care in the community setting the contribution of indirect costs to overall costs increases and that of patient healthcare decreases as disease progresses. As people transition from community care to residential care, the proportion of spending on social care increases and that of indirect costs substantially decreases. Such a transition allows some caregivers to regain independency and rejoin the labor force. The reliance on informal care in the community setting is likely due to the differing availability and organization of social care between countries particularly in the earlier, less dependent stages of AD.

## Electronic supplementary material

Supplement 1. Search strategy applied in MEDLINE (PubMED)

Supplement 2. Country-level percentage contribution of cost components by setting and costing approach

Supplement 3. Weighted mean contribution of cost components depending on the inclusion of supervision time in informal caregiving

*Funding:* This work was funded by Biogen.

*Disclosures:* During the peer review process, Biogen had the opportunity to review and comment on the manuscript. The authors had full editorial control of the manuscript and provided their final approval on all content to be published. The authors hold no financial interests in the sponsoring company.

*Ethical standards:* The analysis reported here is based on previously reported literature. No individual patient data has been collected for this study and no ethics approval was required.
