# Supplementary material for: Major Cost Drivers in Assessing the Economic Burden of Alzheimer's Disease: A Structured, Rapid Review
Source: J Prev Alzheimers Dis. 2021 Apr 24;8(3):362–70. doi: 10.14283/jpad.2021.17 (PMC12280782; doi:10.14283/jpad.2021.17)
Supplement: Supplementary file 1 — Supplement 1. Search strategy applied in MEDLINE (PubMED) [file mmc1.pdf]

Supplement 1. Search strategy applied in MEDLINE (PubMED)

#	Query
1	"mixed dementia"[Title/Abstract] OR "alzheimer*"[Title/Abstract] OR "alzheimer disease"[MeSH Terms] OR "mild cognitive impairment"[Title/Abstract] OR "neurocognitive disorder"[Title/Abstract] OR "senile dementia"[Title/Abstract]
2	"frontotemporal"[Title/Abstract] OR "traumatic brain injury"[Title/Abstract] OR "HIV"[Title/Abstract] OR "human immunodeficiency virus"[Title/Abstract] OR "prion disease*"[Title/Abstract] OR "parkinson*"[Title/Abstract] OR "huntington*"[Title/Abstract] OR "medication induced"[Title/Abstract] OR "substance induced"[Title/Abstract] OR "Creutzfeld-Jakob"[Title/Abstract] OR "corticobasal syndrome"[Title/Abstract] OR "corticobasal degeneration"[Title/Abstract] OR "normal pressure hydrocephalus"[Title/Abstract] OR "posterior cortical atrophy"[Title/Abstract] OR "Korsakoff"[Title/Abstract] OR "multiple sclerosis"[Title/Abstract] OR "Niemann-Pick"[Title/Abstract] OR "progressive supranuclear palsy"[Title/Abstract]
3	#1 NOT #2
4	"wage"[Title/Abstract] OR "income"[Title/Abstract] OR "earning*"[Title/Abstract] OR "salary"[Title/Abstract] OR "salaries"[Title/Abstract] OR "pension*"[Title/Abstract] OR "employment"[MeSH Terms] OR "employed"[Title/Abstract] OR "unemployed"[Title/Abstract] OR "underemployed"[Title/Abstract] OR "employment"[Title/Abstract] OR "unemployment"[Title/Abstract] OR "underemployment"[Title/Abstract] OR "workforce"[Title/Abstract] OR "work-force"[Title/Abstract] OR "labor"[Title/Abstract] OR "labour"[Title/Abstract] OR "vocation*"[Title/Abstract] OR "occupation*"[Title/Abstract] OR "economic*"[Title/Abstract] OR "work loss"[Title/Abstract] OR "work participation"[Title/Abstract] OR "work cessation"[Title/Abstract] OR "work status"[Title/Abstract] OR "job loss"[Title/Abstract] OR "job participation"[Title/Abstract] OR "job cessation"[Title/Abstract] OR "job status"[Title/Abstract] OR "worker loss"[Title/Abstract] OR "worker participation"[Title/Abstract] OR "worker status"[Title/Abstract] OR "workers participation"[Title/Abstract] OR "workers status"[Title/Abstract] OR "employee participation"[Title/Abstract] OR "employee status"[Title/Abstract] OR "leaving work"[Title/Abstract] OR "leaving job"[Title/Abstract] OR "stop working"[Title/Abstract] OR "stopped working"[Title/Abstract] OR "ceased working"[Title/Abstract] OR "salaries and fringe benefits/economics"[MeSH Major Topic] OR "salaries and fringe benefits/statistics and numerical data"[MeSH Major Topic] OR "salaries and fringe benefits/trends"[MeSH Major Topic] OR "pensions/economics"[MeSH Major Topic] OR "pensions/statistics and numerical data"[MeSH Major Topic] OR "pensions/trends"[MeSH Major Topic] OR "income/economics"[MeSH Major Topic] OR "income/statistics and numerical data"[MeSH Major Topic] OR "income/trends"[MeSH Major Topic]
5	("expenditure"[Title/Abstract] NOT "energy"[Title/Abstract]) OR "spending*"[Title/Abstract] OR "payment*"[Title/Abstract]
6	"social insurance"[Title/Abstract] OR "social allowance"[Title/Abstract] OR "social benefit*"[Title/Abstract] OR "social security"[Title/Abstract] OR "welfare"[Title/Abstract] OR "transfer payment*"[Title/Abstract] OR "tax benefit"[Title/Abstract] OR "tax credit"[Title/Abstract] OR "care allowance"[Title/Abstract] OR "carer`s allowance"[Title/Abstract] OR "insurance benefits/economics"[MeSH Major Topic] OR "insurance benefits/statistics and numerical data"[MeSH Major Topic] OR "insurance benefits/trends"[MeSH Major Topic]

7	"disability pension"[Title/Abstract] OR "disability allowance"[Title/Abstract] OR "disability benefit"[Title/Abstract] OR "disability insurance"[Title/Abstract] OR "sickness allowance"[Title/Abstract] OR "sickness absence"[Title/Abstract] OR "sick leave"[Title/Abstract] OR "insurance, disability/economics"[MeSH Major Topic] OR "insurance, disability/statistics and numerical data"[MeSH Major Topic] OR "insurance, disability/trends"[MeSH Major Topic]
8	(((((((((((((((("indirect cost*"[Title/Abstract] OR "society"[Title/Abstract]) OR "societal"[Title/Abstract]) OR "government*"[Title/Abstract]) OR "public economic*"[Title/Abstract]) OR "macroeconomic*"[Title/Abstract]) OR "fiscal"[Title/Abstract]) OR "financial"[Title/Abstract]) OR "productivity"[Title/Abstract]) OR "informal care"[Title/Abstract]) OR "unpaid care"[Title/Abstract]) OR "early retirement"[Title/Abstract]) OR (("retire*"[Title/Abstract] OR "retiring"[Title/Abstract]) AND "early"[Title/Abstract])) OR "household cost*"[Title/Abstract]) OR ((("famil*"[Title/Abstract] OR "carer"[Title/Abstract]) OR "caregiver"[Title/Abstract]) AND "impact"[Title/Abstract])) OR ("lost time"[Title/Abstract] OR "time lost"[Title/Abstract])) OR "cost of illness"[Title/Abstract]) OR "cost of illness"[MeSH Terms]
9	#4 OR #5 OR #6 OR #7 OR #8
10	#3 AND #9
11	((((((((((((("epidemiologic studies" [MeSH:NoExp]) OR (case-control studies [MeSH])) OR (cohort studies [MeSH])) OR (case-control[Title/Abstract])) OR (cohort[Title/Abstract])) OR ("follow-up study"[Title/Abstract] OR "follow-up studies"[Title/Abstract])) OR ("observational study"[Title/Abstract] OR "observational studies"[Title/Abstract])) OR (longitudinal[Title/Abstract])) OR (retrospective*[Title/Abstract])) OR (cross-sectional[Title/Abstract])) OR ("cross-sectional studies"[MeSH Terms])) OR (registry[Title/Abstract])) OR (survey[Title/Abstract])) OR ("case series"[Title/Abstract])
12	(review [Publication type] OR "systematic review" [Publication type] OR "meta-analysis" [Publication Type] OR meta-analys*[Title/Abstract] OR review[Title/Abstract] OR qualitative[Title/Abstract] OR interview*[Title/Abstract] OR "focus group*"[Title/Abstract] OR "study protocol"[Title/Abstract] OR editorial[All fields] OR letter[All fields] OR comment[All fields] OR "case report*"[All fields])
13	#11 NOT #12
14	#10 AND #13
15	(#14) AND (("2010/01/01"[Date - Publication] : "2020/12/31"[Date - Publication]))
16	(#15) AND limit to Humans
