# Supplementary material for: Major Cost Drivers in Assessing the Economic Burden of Alzheimer's Disease: A Structured, Rapid Review
Source: J Prev Alzheimers Dis. 2021 Apr 24;8(3):362–70. doi: 10.14283/jpad.2021.17 (PMC12280782; doi:10.14283/jpad.2021.17)
Supplement: Supplementary file 2 — Supplement 2. Country-level percentage contribution of cost components by setting and costing approach [file mmc2.pdf]

Supplement 2. Country-level percentage contribution of cost components by setting and costing approach

		OPPORTUNITY COSTS													REPLACEMENT COSTS			
		Community				Residential				Community and residential					Community			
		n dyads (studies)	Patient health care	Patient social care	Indirect costs	n dyads (studies)	Patient health care	Patient social care	Indirect costs	n dyads (studies)	Patient health care	Patient social care	Indirect costs	Intangible	n dyads (studies)	Patient health care	Patient social care	Indirect costs
**China**	Overall									3046 (1)	32.5%	15.6%	48.3%	4.1%				
**Germany**	Mild	228 (1)	25.9%	6.6%	67.5%										228 (1)*	29.4%	7.5%	63.1%
	Moderate	157 (1)	20.0%	9.0%	71.0%										157 (1)*	21.2%	9.5%	69.4%
	Severe	167 (1)	13.2%	9.3%	77.5%										167 (1)*	14.6%	10.2%	75.1%
	Overall	824 (2)	18.2%	11.3%	70.6%	123 (1)	9.9%	85.9%	4.0%	395 (1)	14.7%	67.5%	17.8%		552 (1)*	19.5%	9.4%	71.0%
**Spain**	Mild	184 (2)	17.4%	11.8%	70.8%										116 (1)*	29.1%	20.5%	50.4%
	Moderate	386 (3)	16.9%	14.2%	68.9%	26 (1)*	10.5%	83.6%	5.9%	120 (1)*	20.8%	47.1%	32.1%		118 (1)*	15.2%	39.1%	45.8%
	Severe	205 (2)	11.4%	21.7%	66.8%	47 (1)*	8.0%	85.0%	7.0%	106 (1)*	13.5%	63.2%	23.3%		146 (1)*	16.5%	42.4%	41.1%
	Overall	601 (2)	13.9%	18.6%	67.5%	73 (1)*	8.9%	84.5%	6.6%	854 (2)	13.9%	36.2%	49.9%		380 (1)*	18.3%	37.4%	44.3%
**France**	Mild	164 (2)	16.8%	17.0%	66.2%										164 (2)	16.8%	17.0%	66.2%
	Moderate	136 (1)	17.2%	18.1%	64.7%										136 (1)	18.6%	19.6%	61.7%
	Severe	145 (1)	13.0%	13.1%	73.9%										145 (1)	13.9%	13.9%	72.2%
	Overall	472 (2)	15.1%	14.8%	70.1%										472 (2)	15.1%	14.8%	70.1%
**Ireland**	Overall	100 (1)	15.1%	12.1%	72.9%													
**Italy**	Mild	29 (1)	7.8%	10.0%	82.2%										29 (1)*	9.8%	12.6%	77.5%
	Moderate	80 (1)	6.3%	9.6%	84.1%										80 (1)*	7.1%	10.9%	82.0%
	Severe	89 (1)	2.6%	9.6%	87.8%										89 (1)*	3.6%	13.5%	83.0%
	Overall	198 (1)	4.2%	9.7%	86.1%										198 (1)*	5.5%	12.5%	82.0%
**Japan**	Mild	156 (1)|*	17.3%	27.1%	55.6%													
	Moderate	209 (1)*	12.5%	32.4%	55.1%													
	Severe	188 (1)*	9.1%	31.2%	59.8%													
	Overall	553 (1)*	11.9%	30.8%	57.3%													
**Sweden**	Mild	69 (1)*	24.7%	45.3%	30.0%					91 (1)*	14.5%	72.2%	13.3%					
	Moderate	77 (1)*	19.6%	37.2%	43.2%	44 (1)*	5.5%	92.2%	2.4%	212 (2)	8.3%	77.1%	14.6%					
	Severe	29 (1)*	13.6%	47.3%	39.1%	56 (1)*	8.2%	89.6%	2.3%	136 (2)	7.6%	84.1%	8.2%					
	Overall	175 (1)*	19.9%	42.1%	38.0%	100 (1)*	7.0%	90.8%	2.3%	508 (2)	9.7%	77.2%	13.1%					
**UK**	Mild	287 (2)	10.6%	18.5%	71.0%										201 (1)*	10.4%	22.1%	67.5%
	Moderate	260 (2)	9.6%	19.5%	70.9%	22 (1)*	6.1%	84.0%	9.9%	103 (1)*	11.2%	48.6%	40.2%		179 (1)*	9.9%	19.9%	70.2%
	Severe	213 (2)	6.1%	21.1%	72.8%	37 (1)*	7.3%	89.3%	3.4%	104 (1)*	8.3%	58.7%	32.9%		146 (1)*	6.5%	22.1%	71.4%
	Overall	760 (2)	8.5%	19.9%	71.7%	59 (1)*	6.8%	87.2%	6.0%	293 (1)*	11.4%	47.4%	41.1%		526 (1)*	8.6%	21.4%	70.0%
**USA**	MCI	677 (1)	50.9%	9.0%	40.1%										677 (1)	79.9%	14.1%	6.0%
	Mild	721 (2)	36.2%	10.1%	53.7%										650 (1)	65.4%	18.2%	16.4%
	Moderate*	83 (1)	30.4%	11.5%	58.1%	40 (1)	9.6%	81.7%	8.7%	123 (1)	17.3%	55.8%	26.9%					
	Severe*	47 (1)	24.0%	12.4%	63.6%	39 (1)	9.7%	82.9%	7.4%	86 (1)	14.0%	61.6%	24.4%					
	Overall*	201 (1)	29.9%	12.1%	58.0%	79 (1)	9.7%	82.3%	8.1%	280 (1)	18.1%	52.8%	29.0%		201 (1)	17.7%	35.3%	47.0%

UK:United Kingdom. USA: United States of America. MCI: Mild cognitive impairment.

* supervision time not included in the valuation of informal care
