# Supplementary material for: Major Cost Drivers in Assessing the Economic Burden of Alzheimer's Disease: A Structured, Rapid Review
Source: J Prev Alzheimers Dis. 2021 Apr 24;8(3):362–70. doi: 10.14283/jpad.2021.17 (PMC12280782; doi:10.14283/jpad.2021.17)
Supplement: Supplementary file 3 — Supplement 3. Weighted mean contribution of cost components depending on the inclusion of supervision time in informal caregiving [file mmc3.pdf]

Supplement 3. Weighted mean contribution of cost components depending on the inclusion of supervision time in informal caregiving

	**Without supervision time**			**With supervision time**		
	Patient Healthcare	Patient Social care	Informal care	Patient Healthcare	Patient Social care	Informal care
**France**						
mild	22.8%	27.5%	50.9%	15.7%	19.0%	65.2%
moderate	25.0%	26.3%	48.1%	17.2%	18.1%	64.7%
severe	21.5%	21.7%	56.8%	13.0%	13.1%	73.9%
overall	22.9%	24.5%	52.7%	14.9%	15.9%	69.2%
**Germany**						
mild	36.9%	9.4%	53.8%	25.9%	6.6%	67.5%
moderate	27.2%	12.1%	60.7%	20.0%	9.0%	71.0%
severe	20.1%	14.1%	65.7%	13.2%	9.3%	77.5%
overall	25.8%	12.5%	61.6%	17.8%	8.6%	73.6%
**UK**						
mild	11.4%	24.3%	65.6%	8.9%	18.8%	72.3%
moderate	12.0%	24.2%	63.9%	8.7%	17.5%	73.9%
severe	8.1%	27.7%	63.9%	5.6%	19.1%	75.3%
overall	10.3%	25.6%	64.5%	7.5%	18.5%	74.0%
**Italy**						
mild	11.4%	14.6%	74.1%	7.8%	10.0%	82.2%
moderate	8.4%	12.8%	78.8%	6.3%	9.6%	84.1%
severe	3.9%	14.6%	81.5%	2.6%	9.6%	87.8%
overall	6.2%	14.1%	79.7%	4.2%	9.7%	86.1%
**Spain**						
mild	18.0%	12.7%	69.4%	12.5%	8.8%	78.7%
moderate	11.3%	29.2%	59.5%	8.1%	20.9%	71.0%
severe	12.3%	31.7%	56.1%	8.6%	22.3%	69.1%
overall	13.2%	26.9%	59.9%	9.3%	18.9%	71.8%
**Between countries**						
mild	21.6%	18.2%	60.2%	15.6%	13.2%	71.2%
moderate	18.1%	21.1%	60.8%	13.0%	15.2%	71.8%
severe	14.4%	21.8%	63.8%	9.5%	14.4%	76.0%
overall	17.2%	20.7%	62.1%	11.9%	14.4%	73.7%
